# Supplementary material for: Shoe-Integrated Sensor System for Diagnosis of the Concomitant Syndesmotic Injury in Chronic Lateral Ankle Instability: A Prospective Double-Blind Diagnostic Test
Source: Nanomaterials (Basel). 2023 May 4;13(9):1539. doi: 10.3390/nano13091539 (PMC10180214; doi:10.3390/nano13091539)
Supplement: Supplementary file 1 [file nanomaterials-13-01539-s001.zip › nanomaterials-2279147-supplementary.pdf]

Table S1. Peak pressure ratio measured by SISS system and calculated predicted probabilities in 27 CLAI patients.

ID	Disease	HP	HA	ML	M5	M3	M1	T1	LOGREGR_Pred
1	0	0.904376399	1.15458114	0.713068786	0.943664601	1.032783423	0.700444994	1.557543197	0.69409781
2	1	0.802345726	0.852752511	0.771263762	0.97387009	0.656878041	0.626121477	0.304067695	0.98966388
3	0	0.939239289	1.091250473	0.790205096	0.869520817	0.97732204	0.723975103	1.043787614	0.64736477
4	1	0.978784839	1.011898616	0.828674011	0.748602235	0.737243229	1.018070876	0.485736007	0.86137324
5	1	1.07765745	0.894188562	0.597311911	1.044747806	1.045982907	1.179512203	1.484290514	0.78244472
6	1	0.961186212	1.14802057	0.638759891	0.929359839	1.015241452	0.75969124	0.539833582	0.65500446
7	1	0.962053637	1.064412055	0.548516371	0.639224494	1.073699149	0.406042358	1.01807141	0.62841501
8	1	0.986531878	0.705535437	1.176253065	1.030490122	0.896937012	1.159569525	0.912358739	0.47599898
9	0	1.098934745	0.946179274	1.003195855	1.086412927	0.970064557	1.364343218	0.802573154	0.38905980
10	1	0.989939706	0.899106989	0.62549077	0.810730627	0.981101724	0.586301109	1.172388778	0.88425805
11	0	0.950897779	0.544248599	0.878962824	1.013721652	1.004519359	1.118642331	0.95798625	0.50635406
12	0	0.84804154	0.802138685	2.005865381	0.7378201	0.81689527	0.110566906	NA	0.07981630
13	0	0.987752752	1.636057127	0.479076671	0.891554038	1.085951556	0.813737018	0.883902498	0.47415466
14	0	0.99776292	1.185161962	1.3321073	1.018408641	0.969067218	1.280042498	0.73909726	0.05675877
15	0	0.913114642	0.826095452	1.263778076	1.044765729	0.981144239	1.139694923	1.198713309	0.15479330
16	1	1.045093099	0.991741516	1.181919592	0.920010103	0.909201752	0.668564441	1.1323584	0.68792902
17	0	0.959147072	1.204964988	0.895341342	0.839120885	1.009591553	0.823829899	1.123595652	0.33061976
18	1	0.899196417	0.738913144	0.687866876	1.111027194	0.977894751	0.800407337	0.851480078	0.88769999
19	1	1.035220693	1.057507955	0.387726509	0.630295592	0.680596121	0.583339946	0.867641979	0.99695217
20	1	0.949658831	1.258216413	1.135738821	0.980318421	0.846697493	0.878097362	1.205800867	0.66228519
21	0	1.403494425	1.701857631	1.188975752	0.967479299	0.841069414	0.76107797	0.998779697	0.90277709

22	1	1.055997924	1.007478825	1.007657592	1.193814234	1.070703864	0.819669808	0.722414622	0.52566602
23	1	0.936694496	0.971966443	0.846393835	0.688881687	0.963326174	0.912075496	1.385083262	0.37989367
24	1	1.234791795	1.741118447	1.092351637	1.046029283	0.977932257	0.90735962	1.055739031	0.51825922
25	0	0.905863226	1.077043568	1.342952806	1.149373954	1.020507025	0.644619122	1.121016991	0.27765857
26	0	0.828632489	0.466172636	1.280594536	1.024908787	0.988583147	1.038412426	1.221479884	0.17240373
27	1	1.175349749	1.85247218	1.186727762	1.168373132	1.03299746	0.856189289	1.375291247	0.37829778

Disease: 0 = normal syndesmosis; 1 = syndesmotic injury;

LOGREGR_Pred: predicted probability calculated by logistic regression;

NA: not applicable.
